# Multiparametric MRI Markers Associated with Breast Cancer Risk in Women with Dense Breasts

**DOI:** 10.3390/cancers17233771

**Published:** 2025-11-26

**Authors:** Wesley Surento, Romy Fischer, Debosmita Biswas, Daniel S. Hippe, Anum S. Kazerouni, Jin You Kim, Isabella Li, John H. Gennari, Habib Rahbar, Savannah C. Partridge

**Affiliations:** 1 Department of Radiology, University of Washington, Seattle, WA 98195, USA; wsurento@uw.edu (W.S.); romy.fischer.01@gmx.de (R.F.); biswasd@uw.edu (D.B.); anumkaz@uw.edu (A.S.K.); jinyoukim@pusan.ac.kr (J.Y.K.); bellali7@uw.edu (I.L.); hrahbar@uw.edu (H.R.); 2 Department of Biomedical Informatics and Medical Education, University of Washington, Seattle, WA 98195, USA; gennari@uw.edu; 3 Charité—Universitätsmedizin Berlin, 10117 Berlin, Germany; 4 Department of Bioengineering, University of Washington, Seattle, WA 98195, USA; 5 Clinical Research Division, Fred Hutchinson Cancer Center, Seattle, WA 98109, USA; dhippe2@fredhutch.org

**Keywords:** magnetic resonance imaging (MRI), background parenchymal enhancement (BPE), background parenchymal diffusion signal (BPDS), diffusion-weighted imaging, Tyrer–Cuzick risk score

## Abstract

**Background/Objectives**: This study explored the associations of normal breast tissue characteristics on multiparametric MRI with clinical assessments of breast cancer risk in women with dense breasts. **Methods**: Women with dense breasts who underwent multiparametric MRI were included. Breast cancer risk was determined based on Tyrer–Cuzick (TC) lifetime risk scores, categorized as high (TC ≥ 20%) or low risk. Qualitative background parenchymal enhancement (BPE) assessment was obtained from imaging reports. Quantitative imaging markers were calculated, including median BPE, median apparent diffusion coefficient, and volume measures of the whole breast, fibroglandular tissue (FGT), blood vessels, and BPE regions. The associations between imaging markers and TC risk groups were evaluated using age-adjusted logistic regression and summarized by area under the receiver operating characteristic curve (AUC). **Results**: Seventy-seven women were evaluated; a total of 20 (26%) were low risk, and 57 (74%) were high risk. After adjusting for age and multiple testing, BPE:breast ratio (adj. *p* = 0.037), FGT:breast ratio (adj. *p* = 0.046), and BPE:vessel ratio (adj. *p* = 0.037) were positively associated with risk, while qualitative BPE was not (adj. *p* = 0.11). Overall, risk categorizations based on imaging markers were concordant with TC score in up to 70% of women.** Conclusions**: In women with dense breasts, quantitative measures from multiparametric MRI (BPE:breast, FGT:breast, and BPE:vessel ratios) moderately discriminated high- and low-risk groups, warranting further investigation of their value to supplement conventional breast cancer risk assessment tools.

## Simple Summary

Breast cancer risk assessment tools such as the Tyrer–Cuzick model estimate lifetime risk of developing cancer and are designed to help inform additional screening and possibly early interventions. Multiparametric MRI may offer more direct insights into a woman’s breast tissue biology and cancer risk beyond the clinical factors used in the assessment tools. In this study, we explored both qualitative, radiologist-assessed features as well as quantitative imaging features that may offer more comprehensive and reproducible measures of normal breast tissue characteristics. We found that several quantitative MRI markers were significantly associated with Tyrer–Cuzick risk scores, warranting further study of their value to augment risk models to better inform clinical decision making.

## 1. Introduction

Breast cancer remains the most common cancer among women worldwide and second leading cause of cancer-related deaths among women in the United States [1]. One in eight women will develop breast cancer in their lifetime [2], and the incidence continues to rise [3]. As such, early and accurate risk stratification is essential to improve screening management and patient outcomes. Risk assessment tools are commonly used in the clinical setting, with the Tyrer–Cuzick (TC) model being one of the most comprehensive [4], to estimate lifetime risk of developing cancer. This study examines the association between MRI markers and TC-based risk scores for differentiation of breast cancer risk in women with dense breasts, a population for whom mammographic screening is recognized to be insufficient and supplemental screening strategies are often recommended.

It is increasingly recognized that imaging-based biomarkers can play a critical role in breast cancer risk assessment. Among the most established is mammographic breast density, which describes the ratio of fibroglandular tissue (FGT) to fatty tissue and is known to be an independent risk factor for breast cancer [5]. On magnetic resonance imaging (MRI), higher levels of background parenchymal enhancement (BPE) are also associated with increased breast cancer risk, independent of breast density [6,7,8,9,10]. BPE reflects the degree of FGT enhancement on dynamic contrast-enhanced (DCE) imaging and is influenced by underlying hormonal activity and vascularity [11]. While mammographic density and BPE are commonly assessed visually and assigned to one of four categories by the breast radiologist, these qualitative evaluations are subjective and susceptible to inter- and intra-observer variability [12,13].

Quantitative imaging biomarkers, on the other hand, are objectively measured characteristics derived from radiologic images [14]. They may offer more precise predictive value at the individual level and further improve breast cancer risk assessment. In this context, previous studies have demonstrated quantitative BPE measures to have significant potential as predictive markers of breast cancer risk [15,16,17]. Moreover, multiparametric breast MRI exams hold rich biological information beyond BPE, and there is opportunity to derive a wide range of features to characterize breast tissue that are underexplored in relation to breast cancer risk. These include breast composition characteristics of relative volumes of FGT, adipose tissue, and blood vessels reflected by unenhanced anatomical images as well as tissue microstructural characteristics reflected by diffusion-weighted imaging (DWI). With the ultimate goal to improve breast cancer risk stratification, we investigated a variety of quantitative multiparametric MRI characteristics for differentiation of breast cancer risk in women with dense breasts.

## 2. Methods

### 2.1. Study Cohort

In this retrospective study, we performed a secondary analysis of patients enrolled in an IRB-approved research study (NCT03607552) evaluating diffusion-weighted MRI for screening women with dense breasts. Eligible participants had dense breasts based on mammography or MRI (BI-RADS density category C-heterogeneously dense or D-extremely dense). All participants underwent multiparametric breast MRI examinations between November 2019 and May 2022 for indications of either high-risk screening or problem-solving MR (following identification of a suspicious lesion on routine mammography or MR screening) and confirmed to be negative for cancer based on biopsy and 1-year follow-up. At our institution, TC risk assessment is routinely performed as part of the clinical workflow during breast screening. TC lifetime risk scores and clinical factors (age, demographics, menopausal status) were retrospectively extracted from electronic medical records.

### 2.2. Image Acquisition and Interpretation

Multiparametric breast MRI scans were acquired on a Philips Achieva 3T scanner using a dedicated 16-channel breast coil (Mammotrak, Philips Medical Systems, Best, The Netherlands). Dynamic contrast-enhanced (DCE) MRI involved one pre-contrast and multiple post-contrast T1-weighted scans, with the first post-contrast scan centered at 110 s after contrast injection (0.1 mmol/kg body weight; ProHance, Bracco Diagnostics Inc., Milan, Italy). Diffusion-weighted imaging (DWI) was acquired with b-values 0, 100, 800, and 1200 s/mm^2^. The details of the acquisition parameters are in Table 1.

**Figure 1:**
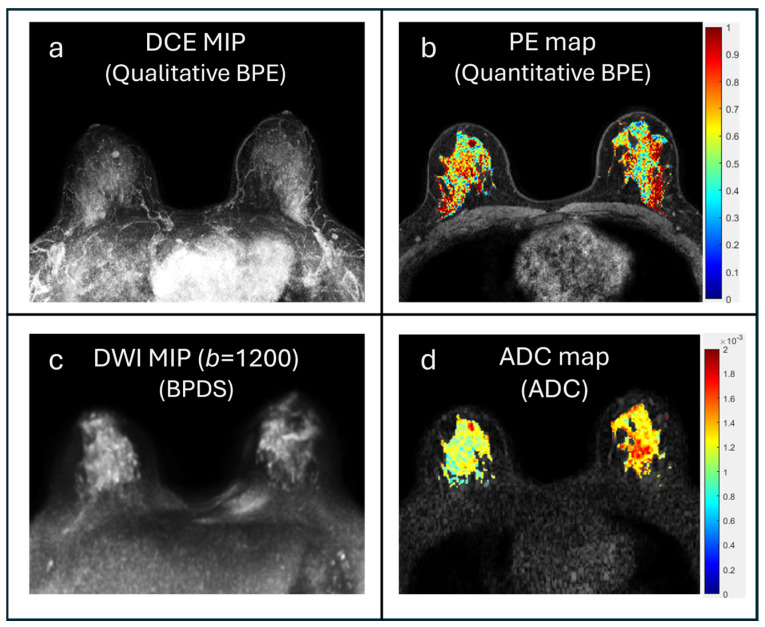
Imaging markers extracted from multiparametric MRI. Examples are shown from a 37-year-old woman with dense breasts, with qualitative BPE assessed as marked ((**a**), DCE MIP shown) by radiologists. Quantitative BPE measures calculated using whole-breast FGT segmentation and PE map (**b**), yielding a median BPE 47.7% and BPE volume of 262 cm^3^. Qualitative BPDS assessed as marked by a radiologist from b = 1200 s/mm^2^ DWI (**c**) and associated ADC map (**d**), with median ADC measured for the whole-breast FGT of 1.41 × 10^−3^ mm^2^/s.

Qualitative BPE assessments were obtained from imaging reports, where BPE was assessed by radiologists using DCE subtraction images (first post-contrast minus pre-contrast) and maximum intensity projections (MIPs) at the time of clinical interpretation, and categorized as minimal, mild, moderate, or marked in accordance with BI-RADS guidelines [18]. Background parenchymal diffusion signal (also known as diffusion background signal [19]), defined as the persistent signal from fibroglandular tissue on DWI, was similarly assessed retrospectively by a radiologist using b = 1200 s/mm^2^ images (BPDS) and categorized as minimal, mild, moderate, or marked, as previously described [18] (Figure 1).

### 2.3. Quantitative Imaging Markers

**Figure 2:**
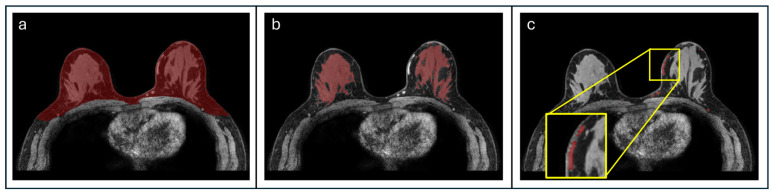
Automated volumetric segmentations. Shown are representative masks (red) generated using a deep learning-based segmentation algorithm by Lew et al. [20] of (**a**) bilateral whole breasts, (**b**) FGT, and (**c**) larger vessels (as seen in the magnified box, yellow).

Quantitative imaging markers were calculated using custom software developed in MATLABR2021b 9.11.0.2358333 Update 7 (Mathworks, Natick, MA, USA). Pre-contrast DCE images underwent preprocessing to correct for patient motion using 2D affine registration and then resampled to isotropic resolution (1 × 1 × 1 mm). Image segmentation was performed using an open source deep learning-based algorithm [20], which reported Dice similarity coefficients of 0.92 for breast, 0.86 for FGT, and 0.65 for blood vessels when compared to manually annotated masks. Our recent evaluations have shown this algorithm to provide accurate segmentations based on radiologist assessment while improving efficiency and reproducibility over semi-automated methods [21]. Using this algorithm, volumetric masks were generated for bilateral whole breasts, FGT, and large, apparent vessels (Figure 2). Masks of the whole breast, FGT, and vessels were used to derive volumetric metrics for the analysis (Table 2).

Percent enhancement (PE) maps were calculated as (S_1_ − S_0_)/S_0_ × 100%, where S_0_ and S_1_ are the pre-contrast and first post-contrast DCE-MR images, respectively. A base threshold (PE > 10%) was applied to exclude sporadic noise, only including voxels that exhibited a PE of greater than 10% for analysis. Quantitative BPE calculations were then performed by applying the FGT mask to the PE map (Figure 1b), further described in Table 2.

**Figure 3:**
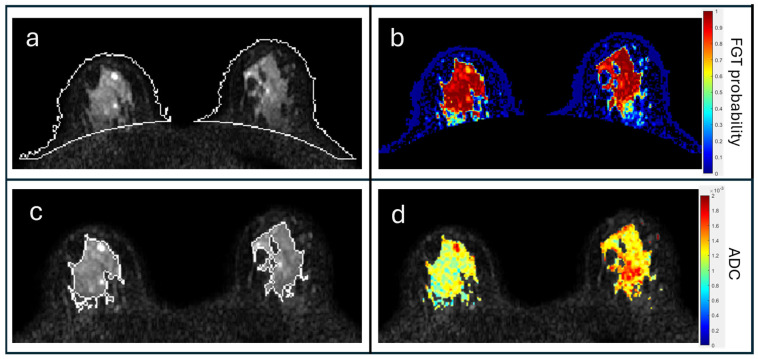
Segmentation of FGT on DWI. Shown are outputs from custom segmentation pipeline using b = 0 DWI, generating whole-breast masks ((**a**), white outline on *b* = 0), fuzzy c-means probability map of FGT (**b**), final FGT mask ((**c**), white outline on *b* = 0), and final ADC map of masked FGT region (**d**).

Apparent diffusion coefficient (ADC) maps were calculated with b = 0, 800 s/mm^2^ using voxel-wise fitting of the monoexponential decay model [22]. Bilateral FGT masks were segmented on b = 0 s/mm^2^ images using a custom semi-automated pipeline developed in MATLAB (Mathworks, Natick, MA, USA) based on fuzzy c-means clustering, first identifying the whole-breast area and then the FGT region (Figure 3). FGT masks were then applied to ADC maps to calculate summary ADC metrics (median, interquartile range [IQR], skewness).

### 2.4. Statistical Analysis

Four subgroups of imaging markers were defined and evaluated: (1) qualitative markers (BPE and BPDS), (2) tissue volume measures (breast volume, FGT volume, vessel volume, FGT:breast ratio, vessel:breast ratio, vessel:FGT ratio), (3) BPE-related measures (median PE, BPE volume, BPE:breast ratio, BPE:FGT ratio, BPE:vessel ratio, integrated intensity), and (4) diffusion-weighted imaging measures (median ADC, ADC IQR, and ADC skewness).

The associations between imaging markers and patient age were evaluated using Spearman’s correlation. The TC score was used to classify patients into low-risk (≤20%) and high-risk groups (>20%). Discrimination between these groups using individual imaging markers was assessed using receiver operating characteristic (ROC) curves and summarized using the area under the ROC curve (AUC). DeLong’s test was used to compare AUC values between markers.

For the primary analysis, logistic regression analysis was conducted to assess the associations of individual imaging markers with TC group after adjusting for age. Histograms were used to visually assess the distributions of markers across patients. Markers demonstrating right-skewness were log-transformed to reduce asymmetry before including them in the models. Associations were summarized using odds ratios (ORs). We adjusted for age in the modeling since it may affect parenchymal tissue biology and enhancement [23].

Due to the number of imaging markers being tested for statistical significance, it was necessary to adjust the *p*-values to reduce the risk of false positive findings in the primary analysis. Holm’s method was used to adjust *p*-values from the age-adjusted logistic regression analysis within each imaging marker subgroup (qualitative markers, segmentation-derived volume measures, BPE-related measures, and diffusion-weighted imaging measures). Adjusted *p*-values (adj. *p* < 0.05) were considered statistically significant in the primary analysis. *p*-values from other analyses were not adjusted, and statistical significance in these analyses was defined as *p* < 0.05 without adjustment.

Concordance rates between actual TC risk group and predicted TC risk group based on the age-adjusted logistic regression models were further investigated. This analysis was limited to the quantitative markers that had a statistically significant association with TC risk group in the primary analysis and qualitative BPE as a reference. The predicted TC risk group was classified as high risk if the predicted probability of being high risk exceeded the prevalence of the TC high-risk group in the whole cohort (74%) and low risk otherwise.

## 3. Results

### 3.1. Patient Characteristics and Follow-Up

Seventy-seven women with dense breasts were identified as eligible and included in the analysis (median age: 45 years, range: 26–71 years), of which 57 (74%) were at high risk of breast cancer. The majority of women were pre- or peri-menopausal (72.7%), had heterogeneously dense breasts (61%), were of White race (82%), and at high risk (74%) (Table 3). The proportion of women with extremely dense breasts was greater in the high-risk group (27/57; 47%) compared to the low-risk group (3/20; 15%), aligning with established associations of density with risk. By most recent follow-up at median 26.4 months (range 17.4 to 45.5 months) after MRI, two patients had developed breast cancer: one with TC lifetime risk of 22.9% who was diagnosed with invasive lobular carcinoma at 25.6 months after MRI and another with TC lifetime risk 47.7% who was diagnosed with invasive ductal carcinoma at 27.4 months after MRI.

### 3.2. Correlation Between Imaging Markers and Age

The imaging markers are summarized in Table 4 with their overall distributions and correlations with age. In general, BPE measures (Spearman’s rho: −0.29 to −0.47) and FGT-associated volume measures (Spearman’s rho: −0.30 to −0.38) were inversely correlated with age (Spearman’s rho: −0.14 to −0.47), while vessel-associated volume measures (Spearman’s rho: 0.29 to 0.38) and breast volume (Spearman’s rho: 0.25) were positively correlated with age. All correlations with age were statistically significant except for median ADC (Spearman’s rho: −0.14, *p* = 0.23).

### 3.3. Association Between Imaging Markers and Tyrer–Cuzick Risk Classifications

**Figure 4:**
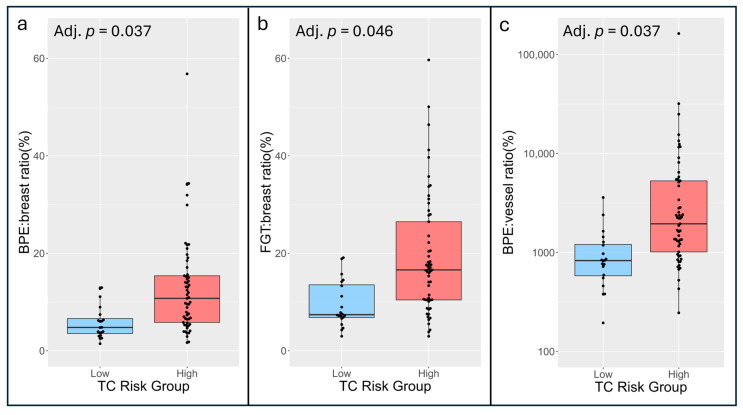
Risk-stratified distributions of select imaging markers. Age-adjusted logistic regression analysis demonstrated significant differentiation between low- and high-risk groups (adjusted *p*-values < 0.05). Imaging markers shown are BPE:breast ratio (**a**), FGT:breast ratio (**b**), and BPE:vessel ratio ((**c**), shown on log_10_ scale).

Qualitative and quantitative imaging markers stratified by TC risk group are shown in Table 5. Qualitative BPE levels and most quantitative volume and BPE measures tended to be higher in the high-risk versus low-risk group, while the opposite trend was observed in vessel-associated measures. After adjusting for age, BPE:breast ratio (OR: 2.71 per 2-fold increase, adj. *p* = 0.037), FGT:breast ratio (OR: 2.59 per 2-fold increase, adj. *p* = 0.046), and BPE:vessel ratio (OR: 4.59 per 2-fold increase, adj. *p* = 0.037) were each individually associated with higher risk based on TC score, as illustrated in Figure 4. Neither qualitative BPE (OR: 1.79 per 1-level increase, adj. *p* = 0.11) nor qualitative BPDS (OR: 1.31 per 1-level increase, adj. *p* = 0.38) were significantly associated with risk group after adjusting for age. No other volume measure (adj. *p* > 0.055), BPE measure (adj. *p* > 0.22), or diffusion-weighted measure (adj. *p* > 0.44) was significantly associated with risk group (Table 5).

**Figure 5:**
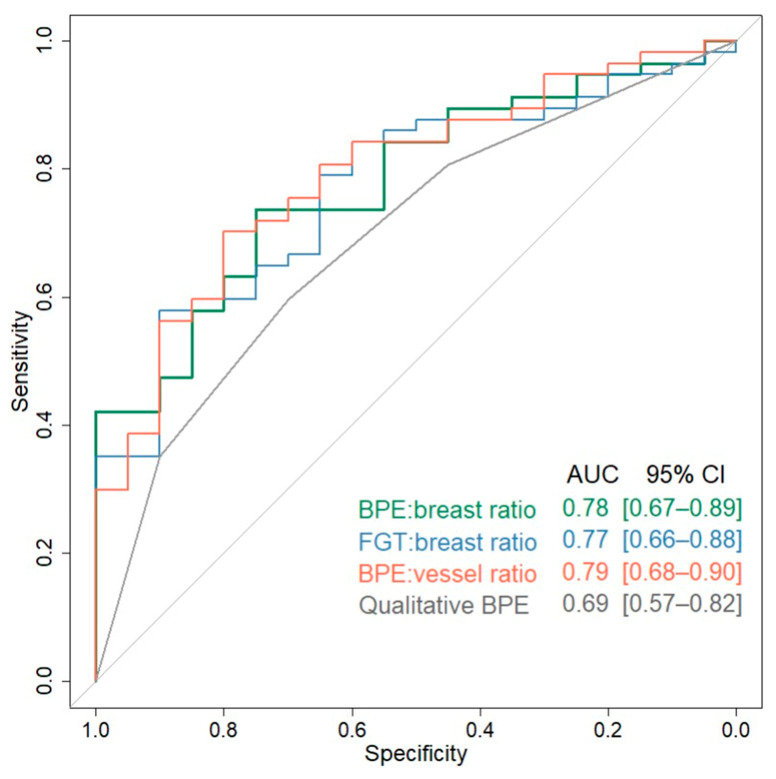
Diagnostic performance of imaging markers. ROC curves for discriminating between low- and high-risk groups for quantitative imaging markers that were statistically significant in the age-adjusted primary analysis (Table 5) as well as qualitative BPE for reference.

ROC curves for the three significant quantitative imaging markers (AUC: 0.77–79) and qualitative BPE (AUC: 0.69) are shown in Figure 5. While these three quantitative markers provided numerically greater discrimination between low- and high-risk groups than qualitative BPE (AUC: 0.69), the AUC values were not statistically significantly different (*p* > 0.22 for each by DeLong’s test).

### 3.4. Concordance Between Imaging Markers and Tyrer–Cuzick Risk Classifications

**Figure 6:**
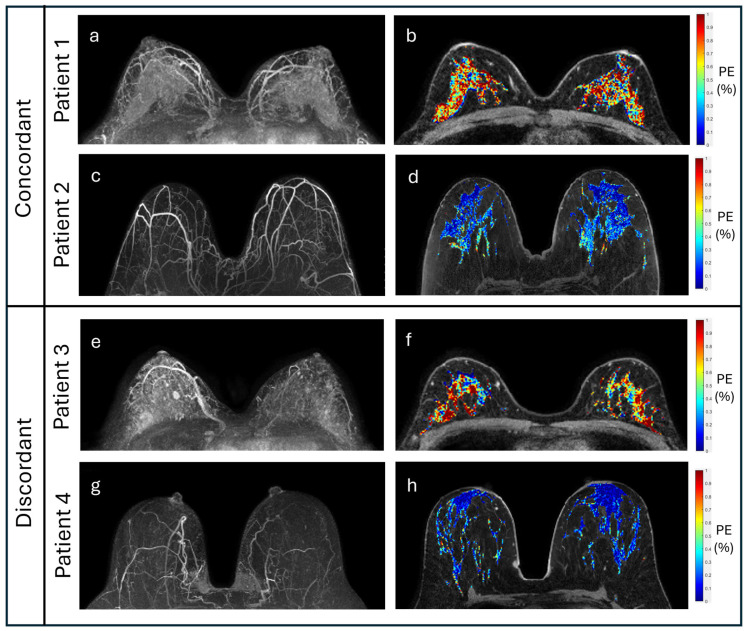
Examples of concordant and discordant cases. Subtraction MIPs (left column) and quantitative PE maps (right column) for several patients with concordant and discordant risk categorizations based on imaging markers and TC score. Patients 1 and 2 are examples of concordant cases. Patient 1 (**a**,**b**) is a 44-year-old woman with high TC risk (30%) due to past history of lobular carcinoma in situ and atypical lobular hyperplasia, exhibiting marked BPE and BPE:breast ratio of 12.5%. Patient 2 (**c**,**d**) is a 69-year-old woman with low TC risk (16%), exhibiting mild BPE and BPE:breast ratio of 3.1%. Patients 3 and 4 are examples of discordant cases. Patient 3 (**e**,**f**) is a 44-year-old woman with low TC risk (18%), exhibiting moderate BPE and BPE:breast ratio of 13.0%. Patient 4 (**g**,**h**) is a 60-year-old woman with high TC risk (27%) due to family history of breast cancer, exhibiting minimal BPE and BPE:breast ratio of 3.6%.

We examined the concordance rates between actual and predicted TC risk group based on the age-adjusted logistic regression models to better understand agreement between MRI markers and Tyrer–Cuzick risk classifications, focusing on qualitative BPE and the statistically significant quantitative markers (Table 6). Across the markers, overall concordance rates reached up to 70% and were similar within low- (65–75%) and high-risk groups (67–70%), representing moderate concordance overall. Figure 6 presents different examples of concordance between PE and TC risk alongside discordance between PE and TC risk.

## 4. Discussion

Our study explored a wide range of breast MRI-derived parameters and found that several quantitative MRI markers incorporating BPE and anatomical volumes could be valuable tools to estimate breast cancer risk. Specifically, we found BPE:breast, FGT:breast, and BPE:vessel volume ratios differentiated between low and high Tyrer–Cuzick risk groups (AUCs: 0.77–0.79) and retained significant association with risk after adjusting for age and multiple comparisons. Qualitative BPE, on the other hand, demonstrated a weaker association with risk (AUC: 0.69). Overall, our work validates the findings from prior studies identifying BPE as a marker of breast cancer risk [6,7,8,9,10,11,13,15,16,17,24,25] and adds new evidence relating to the potential of other alternate quantitative imaging markers that may be associated with risk. Importantly, these quantitative imaging measures may serve as objective biomarkers that could be incorporated into clinical risk assessment algorithms to improve tailoring of screening and preventative interventions based on a more precise estimate of an individual woman’s risk of developing future breast cancer.

Our study further confirms BPE to be associated with a woman’s breast cancer risk. While the exact mechanism by which BPE is associated with breast cancer development remains unclear, it is thought that BPE is a marker of physiologically active tissue responsive to hormonal fluctuations. Higher BPE is caused by an increase in permeability of vessels and uptake of contrast by the fibroglandular tissue in response to the effects of estrogen [26,27]. These highlighted areas of physiologically active breast tissue are perhaps prone to increased inflammation and linked to favorable microenvironments for tumorigenesis [28], therefore elevating cancer risk. The association between increased BPE and cancer risk is reflected in our finding of women who have a higher BPE:breast ratio being nearly three times more likely to be in the high cancer risk group by Tyrer–Cuzick (OR: 2.71, adj. *p* = 0.037). Our observations are consistent with those found by others investigating quantitative measures of BPE, where relative volumes of BPE differentiated women who subsequently developed cancer versus those who did not [15,16].

A novel finding of our study was the strong predictive value of the BPE:vessel ratio for breast cancer risk. The critical role of angiogenesis in breast cancer is well documented [29,30,31,32,33,34], and some studies have identified breast vascularity markers associated with cancer prognosis [24] and recurrence [35]. However, to the best of our knowledge, specific vascular features in the breast preceding primary cancer development have not yet been investigated for their potential link to breast cancer risk. Studies have shown that higher BPE correlates with elevated levels of angiogenesis markers, such as vascular endothelial growth factor (VEGF) [24,36]. Women with fatty breasts have also been reported to exhibit increased breast vessel density [37], which may also be linked to higher body mass index (BMI) and increased age [37,38]. This relationship may be reflected in our observation that patients of the low-risk group, who had lower breast densities compared to the high-risk cohort (15% extremely dense versus 47%, respectively), also tended to have higher vessel volume measures (though not statistically significant). Taken together with prior work, these findings emphasize the potential relevance of vascular changes, not only in tumorigenesis but also in anatomic and structural predispositions that may help stratify breast cancer risk.

Given that BPE is known to be associated with breast cancer risk [7,8,15,17], we were interested to examine the association of BPE and other MRI markers with TC-based risk classification. We used TC as a surrogate for breast cancer risk since it is among the most comprehensive risk assessment models considering a broad selection of risk factors such as family history, hormonal profile, and biopsy results [4], and it is used clinically for risk assessment at our institution. However, TC does not incorporate imaging markers beyond mammographic density, thus motivating our work to examine its association with BPE and other MRI markers. We found overall good agreement between risk classifications based on MRI marker predictions and TC scores, reaching up to 70% concordance. However, lack of concordance in 30% or more of cases also suggests they provide different and potentially complementary information that could be useful in combination to improve accuracy for risk prediction, which is important to explore in future work in study cohorts with known cancer outcomes.

There were several limitations to our study. First, our study cohort included women who were mostly at high risk of breast cancer as well as some who had suspicious findings on mammogram or ultrasound. Also, our sample size was relatively small with a higher proportion of high-risk individuals, which may limit the generalizability of our findings to a broader population of average risk women. Our study defined cancer risk based on Tyrer–Cuzick lifetime risk score as we lacked complete 5-year follow-up and cancer outcomes in our patient cohort. Future larger studies with longer follow-up periods and pathology-confirmed cancer outcomes are needed to confirm associations of imaging markers with cancer risk. Such a study would also potentially demonstrate the incremental value provided by imaging markers and how best to combine them with clinical risk assessments (e.g., TC scores) to maximize predictive accuracy. Qualitative BPE was manually assessed by variable radiologists at the time of clinical interpretation; therefore, its performance as a marker may have been limited by inter-observer variability compared to BPE assessments by a single reader. Lastly, the vessel segmentation masks captured visually apparent vessels and may have excluded smaller vessels contained within FGT regions. Therefore, the resulting vessel segmentation mask should not be considered a comprehensive vascular map.

## 5. Conclusions

Our study investigated the potential value of multiparametric MRI markers for stratifying breast cancer risk in women with dense breasts. The findings demonstrated that several quantitative MRI markers were significantly associated with risk based on TC risk classifications. Quantitative BPE measurements distinguished women at low risk vs. high risk for breast cancer with higher accuracy than radiologists’ qualitative BPE assessments, with the additional advantage of offering more precise and less subjective evaluations. Beyond BPE, we also identified new markers relating to breast vessel volumes that were associated with risk, which may be worth exploring further. As our next step, we plan to perform a larger retrospective study in patients with known cancer outcomes to confirm the predictive value of these MRI markers and assess their incremental value to improve performance of conventional clinical risk assessment models.

## Figures and Tables

**Table 1:** Image acquisition parameters.

Protocol	DCE	DWI
Sequence Type	3D Fast Field Echo	2D single-shot—Echo Planar Imaging
Acquisition plane	axial	axial
Phase encode	R/L	A/P
Field-of-view (mm)	240 × 360	360 × 360
Slice thickness (mm)	1.5	4
Acquisition matrix	480 × 720	200 × 200
In-plane resolution (mm^2^)	0.5 × 0.5	1.8 × 1.8
TR (msec)	5.7	3500
TE (msec)	3	75
Flip angle	10	90
Fat suppression	SPAIR	SPAIR
b values, s/mm^2^		0, 100, 800, 1200
Gadolinium-based contrast agent	gadoteridol (0.1 mmol/kg body weight)	-
Acquisition time	2 min per phase, (1 pre-contrast, 3 post-contrast phases with k0 at approx. 2, 4, and 6 min after contrast injection) <9 min total	3 min

**Table 2:** Quantitative BPE and volume measures.

	Derivation Formula
Volume measures	
Breast volume (cm^3^)	number of voxels in Breast mask × volume per voxel
FGT volume (cm^3^)	number of voxels in FGT mask × volume per voxel
Vessel volume (mm^3^)	number of voxels in Vessel mask × volume per voxel
FGT:breast ratio (%)	(FGT volume/Breast volume) × 100%
Vessel:breast ratio (%)	(Vessel volume/Breast volume) × 100%
Vessel:FGT ratio (%)	(Vessel volume/FGT volume) × 100%
BPE measures	
Median PE (%)	median PE of voxels > 10% within FGT mask
BPE volume (cm^3^)	number of voxels with PE > 10% within FGT mask × volume per voxel
BPE:breast ratio (%)	(BPE volume/Breast volume) × 100%
BPE:FGT ratio (%)	(BPE volume/FGT volume) × 100%
BPE:vessel ratio	(BPE volume/Vessel volume)
Integrated intensity (cm^3^)	BPE volume × mean PE

**Table 3:** Patient characteristics.

Variable	Overall Cohort (*n* = 77)
Age at MRI, years	45 (40, 52)
Menopausal status	
Pre-menopausal	52 (68%)
Peri-menopausal	4 (5%)
Post-menopausal	21 (27%)
Race	
American Indian/Alaska Native	3 (4%)
Asian	5 (76%)
Black or African American	1 (1%)
White	63 (82%)
More Than Once Race	1 (1%)
Unknown/Not Reported	4 (5%)
Breast Density *	
Heterogeneous	47 (61%)
Extreme	30 (39%)
Tyrer–Cuzick risk	
Low (≤20% lifetime risk)	20 (26%)
High (>20% lifetime risk)	57 (74%)

All data reported in count (%) or median (interquartile range). * Density based on MR or mammography.

**Table 4:** Spearman’s correlations of imaging markers with age.

Variable	Median (IQR)	Spearman’s rho vs. Age	*p*-Value
Qualitative metrics			
Qualitative BPE	3 (1, 4)	−0.31	0.006
Qualitative BPDS	2 (1, 3)	−0.39	<0.001
Quantitative metrics			
Volume measures			
Breast volume (cm^3^)	1597 (1108, 2501)	0.25	0.029
FGT volume (cm^3^)	204 (157, 326)	−0.30	0.007
Vessel volume (mm^3^)	13 (12, 14)	0.31	<0.001
FGT:breast ratio (%)	14.2 (7.6, 19.4)	−0.38	<0.001
Vessel:breast ratio (%)	0.5 (0.4, 0.7)	0.29	0.011
Vessel:FGT ratio (%)	4.0 (1.8, 7.2)	0.38	<0.001
BPE measures			
Median PE (%)	28.1 (23.7, 36.1)	−0.40	<0.001
BPE volume (cm^3^)	147 (85, 262)	−0.36	<0.001
BPE:breast ratio (%)	7.9 (4.8, 14.0)	−0.47	< 0.001
BPE:FGT ratio (%)	62.0 (54.7, 72.9)	−0.29	0.011
BPE:vessel ratio	13.7 (8.3, 28.5)	−0.47	<0.001
Integrated intensity (cm^3^)	49 (26, 105)	−0.43	<0.001
Diffusion-weighted measures			
Median ADC (×10^−3^ mm^2^/s)	1.61 (1.43, 1.77)	−0.14	0.23
ADC interquartile range (×10^−3^ mm^2^/s)	0.49 (0.44, 0.57)	0.24	0.040
ADC skewness (mm^2^/s)	0.03 (−0.27, 0.32)	−0.26	0.022

IQR = interquartile range.

**Table 5:** Association of each imaging marker with cancer risk group.

	Tyrer–Cuzick Risk Group *				
Variable	Low Risk (*n* = 20)	High Risk (*n* = 57)	AUC (95% CI)	Adjusted OR ^†^ (95% CI)	Adjusted *p*-Value ^‡^
Qualitative markers					
Qualitative BPE	2 (1, 3)	3 (2, 4)	0.69 (0.57–0.82)	1.79 (1.00–3.39)	0.11
Qualitative BPDS	2 (1, 2)	2 (1, 3)	0.63 (0.50–0.76)	1.31 (0.72–2.50)	0.38
Quantitative markers					
Volume measures					
Breast volume (cm^3^)	1918 (1355, 2833)	1516 (1052, 2370)	0.62 (0.49–0.76)	0.74 (0.42–1.27)	0.28
FGT volume (cm^3^)	178 (133, 204)	254 (164, 376)	0.70 (0.58–0.83)	1.83 (1.01–3.52)	0.22
Vessel volume (mm^3^)	11 (8, 15)	7 (4, 14)	0.66 (0.53–0.79)	0.53 (0.24–1.02)	0.24
FGT:breast ratio (%)	7.4 (6.8, 13.5)	16.6 (10.4, 26.5)	0.77 (0.66–0.88)	2.59 (1.34–5.56)	**0.046**
Vessel:breast ratio (%)	0.6 (0.5, 0.7)	0.5 (0.3, 0.6)	0.68 (0.55–0.80)	0.44 (0.15–0.97)	0.24
Vessel:FGT ratio (%)	7.1 (4.6, 9.3)	3.6 (1.3, 6.1)	0.76 (0.65–0.87)	0.28 (0.09–0.67)	0.055
BPE measures					
Median PE (%)	25.9 (23.7, 38.0)	29.2 (23.7, 34.7)	0.55 (0.39–0.71)	0.91 (0.52–1.64)	>0.9
BPE volume (cm^3^)	97 (64, 133)	161 (94, 275)	0.72 (0.60–0.85)	1.81 (1.00–3.46)	0.22
BPE:breast ratio (%)	4.8 (3.5, 6.6)	10.7 (5.8, 15.4)	0.78 (0.67–0.89)	2.71 (1.37–5.98)	**0.037**
BPE:FGT ratio (%)	59.3 (53.7, 68.0)	62.3 (55.5, 75.0)	0.59 (0.45–0.74)	1.19 (0.68–2.06)	>0.9
BPE:vessel ratio	8.3 (5.8, 12.1)	19.5 (10.2, 53.0)	0.79 (0.68–0.90)	4.59 (1.75–15.79)	**0.037**
Integrated intensity (cm^3^)	36 (20, 49)	63 (31, 123)	0.66 (0.52–0.80)	0.69 (0.22–1.15)	0.66
Diffusion-weighted measures					
Median ADC (× 10^−3^ mm^2^/s)	1.56 (1.38, 1.66)	1.61 (1.44, 1.79)	0.61 (0.47–0.76)	1.34 (0.79–2.37)	0.57
ADC interquartile range (× 10^−3^ mm^2^/s)	0.48 (0.44, 0.56)	0.49 (0.44, 0.57)	0.51 (0.36–0.66)	1.32 (0.73–2.58)	0.57
ADC skewness (mm^2^/s)	0.12 (−0.07, 0.33)	−0.06 (−0.31, 0.30)	0.59 (0.45–0.73)	0.66 (0.37–1.15)	0.44

AUC = area under the curve; CI = confidence interval; OR = odds ratio; * Values are bilateral and given as median (interquartile range).^†^ Odds ratios are adjusted for age and expressed as the change per 1-level change (qualitative BPE and BPDS) or per 2-fold change (all quantitative markers).^‡^ *p*-values were adjusted using Holm’s method within subgroups of markers as indicated by the table subheadings. Bold indicates statistical significance after adjustment (adjusted *p* < 0.05).

**Table 6:** Concordance rates of select quantitative markers and qualitative BPE.

		Tyrer–Cuzick Risk Group	
Marker	Overall Cohort (*n* = 77)	Low Risk (*n* = 20)	High Risk (*n* = 57)
BPE:breast ratio	52 (67%)	14 (70%)	38 (67%)
FGT:breast ratio	54 (70%)	14 (70%)	40 (70%)
BPE:vessel ratio	53 (69%)	15 (75%)	38 (67%)
Qualitative BPE	52 (67%)	13 (65%)	39 (68%)

Values are number (%) of cases where the actual and predicted risk groups are equal.

## Data Availability

The data presented in this study are available on request from the corresponding author due to privacy restrictions around protected health information.
